# Investigation of Comorbidity and Risk Factors Analysis During Lumpy Skin Disease Outbreaks in India

**DOI:** 10.3390/microorganisms13030472

**Published:** 2025-02-20

**Authors:** Gundallahalli Bayyappa Manjunatha Reddy, Shraddha Bijalwan, Siju Susan Jacob, Sunil Tadakod, Snigdha Madhaba Maharana, Sudeep Nagaraj, Sai Mounica Pabbineedi, Chandana Ramesh Uma, Viveka Prabhu Balappa, Chethan Kumar Harlipura Basavarajappa, Pinaki Prasad Sengupta, Sharanagouda Shiddanagouda Patil, Baldev Raj Gulati

**Affiliations:** 1 ICAR-National Institute of Veterinary Epidemiology and Disease Informatics (NIVEDI), P.O. Box 6450, Yelahanka, Bengaluru 560119, Karnataka, India; shraddhabijalwan1998@gmail.com (S.B.); drsijunivedi@gmail.com (S.S.J.); sunilnivedi@gmail.com (S.T.); madhabamaharana192@gmail.com (S.M.M.); sudeepcellculture@gmail.com (S.N.); chandanaru27999@gmail.com (C.R.U.); baluvivekprabhu@gmail.com (V.P.B.); chethuhb@gmail.com (C.K.H.B.); pinaki.sengupta@icar.gov.in (P.P.S.); sharanspin13@gmail.com (S.S.P.); brgulati@gmail.com (B.R.G.); 2 Veterinary Pathobiology, Oklahoma Center for Respiratory and Infectious Diseases (OCRID), Oklahoma State University, 250 McElroy Hall, Stillwater, OK 74078, USA; mounica20sai@gmail.com

**Keywords:** comorbidity, haemoprotozoan diseases, infectious bovine rhinotracheitis, lumpy skin disease, malignant catarrhal fever, risk factors, theileriosis

## Abstract

Lumpy skin disease (LSD) is a re-emerging viral transboundary disease affecting cattle and buffaloes, resulting in a significant socio-economic impact on the affected regions. LSD is primarily transmitted among susceptible livestock through hematophagous vectors, including ticks and flies. Ticks also function as reservoirs for various haemoprotozoan parasites, increasing the likelihood of coinfections in affected animals. This study investigates the comorbidity of LSD and associated risk factors using diverse datasets. A total of 414 samples from LSD-suspected animals were screened for LSD, infectious bovine rhinotracheitis (IBR), malignant catarrhal fever (MCF), babesiosis, and theileriosis (*Theileria annulata* and *Theileria orientalis*), as well as anaplasmosis. Among these, 214 (51.6%) tested positive for LSD. A strong correlation was identified between LSD and oriental theileriosis caused by *Theileria orientalis* (50.9%). Other significant associations were observed with IBR (34.1%), anaplasmosis (24.7%), tropical theileriosis (15.4%), babesiosis (12.6%), and MCF (12.1%). The transmission dynamics of LSD revealed that hematophagous vectors, particularly *Stomoxys*, *Haematobia*, and *Rhipicephalus*, play a crucial role in its spread, especially in unorganised farming systems. Additionally, *Haematobia* and *Stomoxys* flies were implicated in the high transmission rate of oriental theileriosis (39%) in conjunction with LSD. Notably, ticks (*Rhipicephalus*) facilitated the concurrent transmission of one, two, or three infections alongside LSD. While Musca, a non-hematophagous fly, was found to carry LSD virus (LSDV), it did not test positive for other pathogens. This study highlights the potential for cattle to harbour multiple diseases simultaneously with LSD, emphasising the necessity for integrated transmission studies and comprehensive disease screening in affected livestock. These findings underscore the importance of implementing targeted prevention and control strategies to mitigate disease impact in livestock populations.

## 1. Introduction

According to the Food and Agriculture Organization (FAO), transboundary animal diseases (TADs) are characterised by their capacity to rapidly spread to new areas and regions, posing a threat to the economic well-being of affected areas. TADs (such as lumpy skin disease (LSD), Peste des petits ruminants (PPR), African swine fever (ASF), Classical swine fever (CSF), etc., are considered notifiable diseases by the OIE due to their impact on the food security of the country [1]. LSD is a highly contagious viral infection that affects bovines, including cattle and water buffalo. Recently, cases of LSD have been reported in Mithun [2] and Yak in India [3]. The LSD virus (LSDV), responsible for this disease, belongs to the Capripoxvirus genus within the Poxviridae family. It has a 151-kilobase pair double-stranded DNA genome and is structurally characterised as an ovoid, linear virion with an envelope [3]. LSD traces its origins back to 1929 in North Rhodesia, now Zambia, located in south–central Africa [4]. In India, the first reported cases emerged in August 2019, specifically in Mayurbhanj, Bhadrak, and other districts of Odisha [5]. LSD is clinically characterised by the presence of nodules throughout the body, along with lacrimal (tear) and nasal discharges. Affected animals commonly exhibit symptoms, such as fever, loss of appetite (anorexia), depression, and reluctance to move. These clinical signs collectively contribute to the overall manifestation of LSD, making it identifiable through a combination of visible skin lesions and observed behavioural and physiological changes in infected cattle. The impact of LSD extends beyond its visible symptoms [3]. LSD induces leukopenia, reducing the white blood cell count and causing immunosuppression, which makes animals more susceptible to other infections and leads to comorbidities, thereby impacting not only the skin but also the overall health and disease resistance of affected animals [6]. Comorbidity refers to the simultaneous presence of two or more diseases in an animal. In cattle, comorbidity can occur with other viral infections, such as infectious bovine rhinotracheitis (IBR) and malignant catarrhal fever (MCF) coexisting with LSD. The concurrent presence of these infections complicates the clinical picture, making diagnosis, treatment, and disease control more challenging in affected cattle populations. Both infectious bovine rhinotracheitis (IBR) and malignant catarrhal fever (MCF), highly contagious viral infections within the Herpesviridae family, have significant economic implications. They can lead to reduced milk production and an increased risk of abortion in affected animals, thereby adversely impacting the cattle industry.

Understanding the transmission dynamics of a disease is crucial to appreciate its epidemiology and broader impact. For LSD, transmission occurs through hematophagous arthropod vectors, primarily flies and ticks. The LSDV is mechanically transmitted by blood-sucking flies, like the common stable fly (*Stomoxys calcitrans*), the Horn Fly (*Haematobia irritants*), and the *Aedes aegypti* mosquito [7,8]. Moreover, ticks such as *Rhipicephalus appendiculatus* and *Amblyomma hebraeum* have been identified as capable vectors of LSDV [9,10]. Ticks and biting flies not only transmit LSDV but also serve as vectors for other pathogens, including haemoprotozoan parasites like *Babesia* spp., *Anaplasma* spp., and *Theileria* spp. This dual role raises the possibility of coinfection in cattle through vectors, where animals could be simultaneously affected by LSD and other vector-borne pathogens. Recent studies have also shown that non-biting flies, such as *Musca domestica*, can carry LSDV. The presence of multiple pathogens can complicate the clinical picture, potentially leading to more severe health consequences in the infected cattle. In addition, the expansion of vector habitats due to the global climate change exacerbates the distribution of pathogens transmitted by vectors [11].

Bovine babesiosis is a haemoprotozoan parasitic disease transmitted by ticks and caused by *Babesia* spp. with a global prevalence of 29%. This disease is a significant concern in the livestock sector, particularly in temperate to tropical countries [12]. Bovine anaplasmosis is caused by the rickettsial organism *Anaplasma marginale*. This intra-erythrocytic pathogen is transmitted biologically by infected ticks or mechanically by biting flies, like *Stomoxys*. The global prevalence of bovine anaplasmosis is reported to be 21% [13]. Bovine theileriosis is a vector-borne disease caused by the haemoprotozoans like *Theileria annulata* and *T. orientalis* in India. Vectors like *Haematobia* and *Stomoxys* have also been implicated in the transmission of theileriosis [14]. Malignant catarrhal fever (MCF) virus belongs to the Herpesviridae family and primarily affects members of the Bovidae family (cattle, bison, and sheep) and the Cervidae family (deer), with occasional cases in the Giraffidae family (giraffes). In MCF, sheep serve as reservoirs for the disease and can transmit it to susceptible hosts, including cattle, water buffalo, bison, deer, and, rarely, giraffes [15].

According to official data, the overall economic impact of LSD during the 2022 outbreak in India was substantial, with estimated losses amounting to INR 17,461.49 crores for affected animals and INR 876.26 crores for deceased animals. Consequently, the total economic loss attributed to LSD reached INR 18,337.76 crores (equivalent to USD 2217.26 million) at the national level [16,17]. These figures underscore the considerable economic consequences of LSD outbreaks, emphasising the need for effective control measures and preventive strategies. The current study focuses on elucidating the coexistence of diseases with LSD and its transmission while considering risk factors, such as age, breed, gender, and geographical region. The aim is to better understand the relationships and patterns of comorbidity associated with LSD across diverse factors in the populations studied.

## 2. Material and Methods

### 2.1. Study Area

During the various outbreaks of LSD in India during 2020–2024, the blood samples were collected from the suspected LSD cases. These samples represented five regions of the country and were collected from eight states of India, such as Karnataka (Southern region), Puducherry (Southern region), Madhya Pradesh (Central region), Chhattisgarh (Central region), Maharashtra (Western region), Gujarat (Western region), Sikkim (Northeastern region), and Himachal Pradesh (Northern region).

### 2.2. Sample Size and Study Design

**Figure 1:**
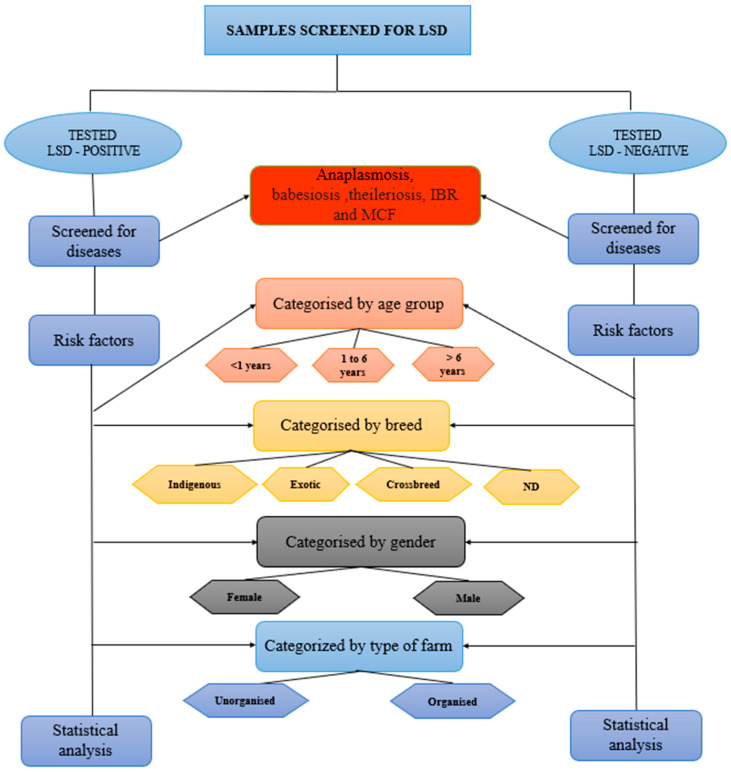
Study design. The schematic diagram representing the processing for the detection of various pathogens in the clinical samples collected from cattle.

A total of 414 blood samples were collected from eight states in India for analysis. A cross-sectional study was conducted to examine the presence of other infectious agents alongside LSDV in the sampled animals. This study employed a questionnaire to retrieve the basic information of the sampled animals including age, sex, breed, and farm type, as well as demographic information, such as state, region, and district. The samples were categorised based on different criteria (Figure 1).

First, age-based classification included less than 1 year (n = 134), 1 to 5 years (n = 147), and more than 5 years (n = 133). Second, gender-based categorisation divided the samples into male (n = 205) and female (n = 209). Third, breed classification included indigenous (n = 122), exotic (n = 103), crossbreeds (n = 97), and non-descript (n = 92). Additionally, the samples were grouped by farm type into unorganised (n = 214) and organised (n = 200).

For transmission studies, vectors were collected, and various parameters were analysed alongside other infectious pathogens alongside LSD. The data collected included farm type (organised or unorganised), vector type (flies or ticks), feeding nature (hematophagous or non-hematophagous), and different vector species—flies (*Musca*, *Haematobia*, and *Stomoxys*) and ticks (*Rhipicephalus* and *Hyalomma*).

### 2.3. Sample Collection and Ethical Statement

With permission from the Department of Animal Husbandry and Veterinary Services of each state, blood samples of 2 to 3 mL were collected from the jugular vein of the cattle in ethylene diamine tetra acetic acid (EDTA) vacutainers. Prior consent from the cattle’s owners was taken, and the data for each sampled cattle were collected by a local veterinarian. The blood samples were then transported in insulated shipping containers with proper cold chain maintenance in dry-ice packs and analysed at the Indian Council of Agricultural Research-National Institute of Veterinary Epidemiology and Disease Informatics (ICAR-NIVEDI), Bengaluru. This study on animals was approved by the Institute Animal Ethics Committee (No. NIVEDI/IAEC/2022/06).

Traps were set for a minimum of 48 h to collect vectors such as *Musca domestica*, *Haematobia*, and *Stomoxys* from various regional outbreak areas. The ticks were manually collected from the animals. In the parasitology laboratory, the vectors were identified using a stereo microscope (Nikon C-LEDS). The tick samples were identified morphologically based on characteristics, such as palps, colour patterns, eyes, festoons, anal grooves, adanal plates, and legs. The fly samples were identified based on the features, like wing venation, aristae, mouthparts, abdominal markings, and segmentation. All the samples were subsequently stored at −20 °C for further analysis.

### 2.4. Sample Processing

From each sample, 150 µL of blood was suspended in 50 µL of phosphate-buffered saline with pH 7.2, while the vector samples were homogenised in 1 mL of 1X PBS. The total genomic DNA was then extracted from the whole blood and vector as per the manufacturer’s protocol using the DNeasy Blood & Tissue kit (QIAGEN, Hilden, Germany). Finally, 30 µL of DNA was eluted in elution buffer. The DNA concentration was determined using a Nanodrop (Nabi, Republic of Korea), measuring the absorbance at the ratio of 260/280 nm, and was stored at −20 °C until further use.

### 2.5. Polymerase Chain Rection (PCR)

A total of 414 blood samples and 100 vector samples were initially tested for lumpy skin disease virus (LSDV) using PCR with a partial p32 primer pair. Subsequently, these samples were further analysed for two viral infections—infectious bovine rhinotracheitis (IBR) and malignant catarrhal fever (MCF)—as well as four haemoprotozoan parasitic diseases, including babesiosis, theileriosis (*Theileria annulata* and *Theileria orientalis*), and anaplasmosis, utilising various primer sets detailed in Table 1. Based on the PCR results, the samples were then classified as LSD-positive or LSD-negative. The PCR reaction conditions are as follows: initial denaturation at 95 °C for 10 min, followed by 34 cycles of denaturation at 95 °C for 30 s, annealing for each primer for 1 min as is mentioned in Table 1, extension at 72 °C for 45 s, and a final extension step at 72 °C for 10 min. A total volume of 25 µL PCR reaction mixture was prepared, comprising 12.5 μL of PCR master mix (Dream Taq Green PCR Master, Thermo Scientific, Waltham, MA, USA), 1 μL each of forward and reverse primers (10 pmol/μL) for each pathogen, 8.5 μL of nuclease-free water, and 50 ng of template DNA. The PCR assays were conducted using a thermal cycler (Bio-Rad, Hercules, CA, USA). Subsequently, the PCR products were subjected to agarose gel electrophoresis on a 1.5% agarose gel in 1X TAE buffer stained with ethidium bromide and docked in a UV transilluminator.

### 2.6. Statistical Analysis

The statistical analysis was performed using the Statistical Package for the Social Sciences (IBM^®^ SPSS^®^ version 27). In this study, lumpy skin disease (LSD), babesiosis, theileriosis (*Theileria annulata* and *Theileria orientalis*), anaplasmosis, infectious bovine rhinotracheitis (IBR), and malignant catarrhal fever (MCF) were designated as dependent variables, as they represented the primary outcomes of interest. Several risk factors, including breed, sex, age, farm type, and vector presence, were considered independent variables (predictors), as they may influence or be associated with the dependent variables.

A univariate analysis was initially performed to assess each risk factor individually. Following this, a multivariate logistic regression analysis was conducted to evaluate the combined effect of multiple risk factors.

In logistic regression, the odds ratio (Exp(B)) was calculated to determine the strength and direction of the association between the predictor variables and disease outcomes. The odds ratio is derived using the following formula:

$$Exp\left(B\right)=e^{B}$$

where

B represents the logistic regression coefficient (extracted from SPSS output);

e denotes Euler’s number.

A 95% confidence interval (CI) was also estimated to provide a range within which the true population parameter is likely to fall, helping quantify uncertainty and assess the precision of the estimates. The CI limits were calculated as follows:

$$Lower\;CI=e^{B-\left(1.96\times S.E\right)}$$

$$Upper\;CI=e^{B+(1.96\times S.E)}$$

where

B is the logistic regression coefficient;

S.E represents the standard error of B (from SPSS data);

e is Euler’s number.

Here, 1.96 is the critical value corresponding to a 95% confidence level in a normal distribution.

Statistical significance was assessed to determine whether a risk factor was significantly associated with the disease outcomes. A *p*-value < 0.05 indicated statistical significance, suggesting that the observed effect was unlikely to be due to random variation, whereas a *p*-value ≥ 0.05 suggested no significant association.

In logistic regression, the Wald test was employed to assess the statistical significance of each predictor variable by determining whether its regression coefficient (*B*) significantly differed from zero. The Wald statistic (*W*) was computed as follows:

$$W=\frac{B^{2}}{{S.E}^{2}}$$

where

*B* represents the logistic regression coefficient;

*S.E* is the standard error of B.

The corresponding *p*-value was derived by comparing the Wald statistic to a chi-square (χ^2^) distribution with one degree of freedom.

An alternative method used included applying syntax for the regression analysis. The syntax in the file dialog box in the New section (syntax) is as follows:

For the univariate analysis:

LOGISTIC REGRESSION VARIABLES dependent variable

  /METHOD = ENTER individual covariate variables/independent variable

  /CONTRAST (1st covariate) = Indicator

  /PRINT = GOODFIT CI (95)

  /CRITERIA = PIN (0.05) POUT (0.10) ITERATE (20) CUT (0.5).

For the multivariate analysis:

LOGISTIC REGRESSION VARIABLES dependent variable

  /METHOD = ENTER all covariate variables/independent variable

  /CONTRAST (1st covariate) = Indicator

  /CONTRAST (2nd covariate) = Indicator

  /CONTRAST (3rd covariate) = Indicator

  /CONTRAST (4th covariate) = Indicator

  /PRINT = GOODFIT CI (95)

  /CRITERIA = PIN (0.05) POUT (0.10) ITERATE (20) CUT (0.5).

A chi-square analysis was employed using GraphPad Prism 8.0.1. The chi-square test with a positive percentage is employed in this study to analyse the categorical data and determine whether there is a significant association between different variables and the proportion of positive cases.

The chi-square was calculated using the following formula:

$$x^{2}=\sum \frac{{\left(O-E\right)}^{2}}{E}$$

where

*O* = the observed frequency in each category.

*E* = the expected frequency in each category.

Σ = the summation across all the categories.

## 3. Results

### 3.1. Epidemiology of LSD in India

**Figure 2:**
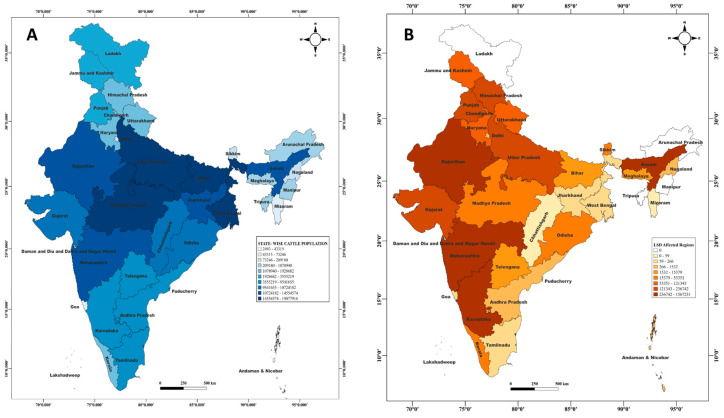
Demographic distribution. Map showing density-wise distribution of (**A**) cattle population shown in gradient of blue and (**B**) LSD-affected regions in gradient of brown in different states of India.

According to the Basic Animal Husbandry Statistics (BAHS) and the 20th Livestock Census, the central and northern regions of India, including Madhya Pradesh, Rajasthan, and Bihar, exhibit a high cattle population. In contrast, the southern and western regions, such as Maharashtra, Gujarat, Andhra Pradesh, and Karnataka, have a moderate cattle population (Figure 2A).

According to the Department of Animal Husbandry and Dairying (DAHD) [25], during the year 2022–2023 LSD outbreak, Himachal Pradesh recorded the highest morbidity rate at 8.1%. Karnataka exhibited the highest mortality rate at 9.7%, followed by Maharashtra (8.5%) and Himachal Pradesh (8.1%). Gujarat reported a morbidity rate of 1.83% and a mortality rate of 3.52%, while Madhya Pradesh recorded morbidity and mortality rates of 0.19% and 2.07%, respectively. Other states (OSs), including Chhattisgarh and eight additional states, reported lower morbidity (0.04%) and mortality (1.31%) rates. The Northeastern region (NER), comprising six states, exhibited a combined morbidity rate of 2.18% and a notably high mortality rate of 20.99%.

### 3.2. Molecular Detection of the Pathogens by PCR

Out of a total of 414 samples screened for LSD, 214 (51.6%) tested positive, indicating a high prevalence of the disease among the sampled animals. The remaining 200 animals (48.3%) were found to be negative for LSD by PCR.

**Figure 3:**
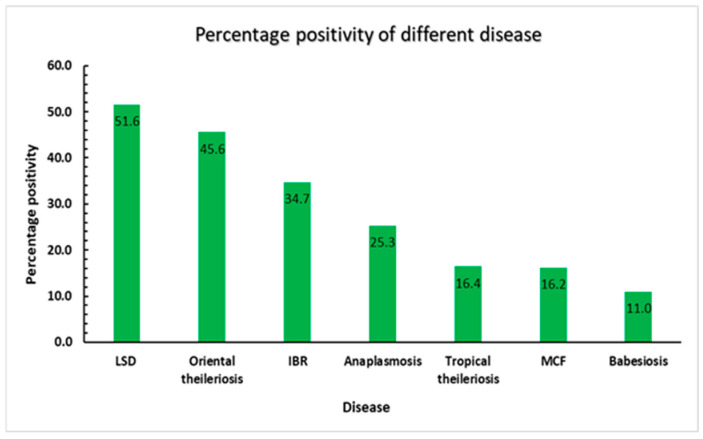
Distribution of different diseases in cattle. The bar diagram representing the percentage positivity of LSD, IBR, MCF, babesiosis, anaplasmosis, oriental theileriosis, and tropical theileriosis on the X-axis and the percentage positivity on the Y-axis.

All 414 samples were analysed for two viral diseases and four haemoprotozoan infections. Among these, babesiosis was detected in 48 animals (10.95%), confirming the presence of the infection in the study regions, though its prevalence was lower than that of LSD. Anaplasmosis was identified in 111 animals (25.3%), affecting approximately one-quarter of the tested population. Oriental theileriosis, caused by *Theileria orientalis*, was detected in 200 animals (45.6%), indicating a widespread distribution of the pathogen across the study areas. Bovine tropical theileriosis, caused by *Theileria annulata*, was found in 72 animals (16.43%), suggesting a moderate occurrence. Infectious bovine rhinotracheitis (IBR) was detected in 152 animals (34.7%), affecting more than one-quarter of the sampled population. Lastly, malignant catarrhal fever (MCF) was identified in 71 animals (16.2%), exhibiting a lower prevalence compared to the other diseases tested (Figure 3).

Of the 100 vectors, 54% were flies, and 46% were ticks, all of which were screened for LSDV and other diseases. None of the screened fly samples tested positive for IBR, MCF, babesiosis, or anaplasmosis. However, all the screened *Stomoxys* and *Haematobia* demonstrated the presence of both LSDV and *T. orientalis*, while *Musca domestica* showed the presence of LSDV. Among the ticks, *Hyalomma* tested negative for LSDV and other pathogens. In contrast, *Rhipicephalus* showed the presence of LSDV, *Anaplasma* spp., *Babesia* spp., and *T. orientalis* but tested negative for IBR and MCF.

### 3.3. LSD and Its Associated Risk Factors

**Figure 4:**
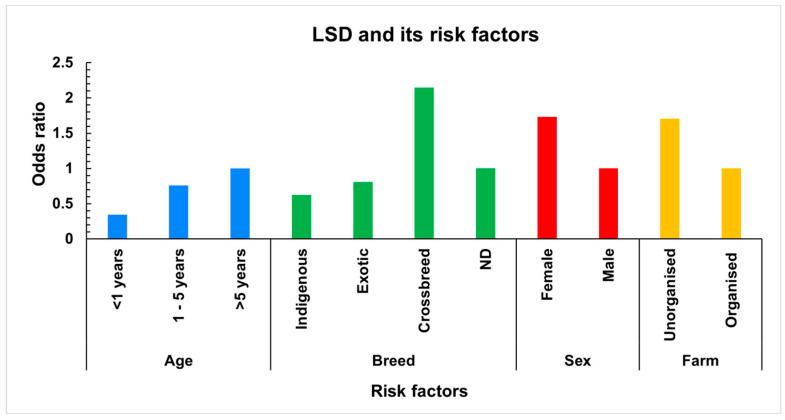
LSD risk factors. The bar diagram showing the association of risk factors (age, breed, sex, and type of farm) with occurrence of lumpy skin disease using univariate regression analysis.

The univariate and multivariate regression analyses identified several risk factors associated with the incidence of LSD, with the calculation of the odds ratios (ORs) revealing significant findings (Figure 4 and Table 2). Age was a critical determinant, with animals aged over 5 years exhibiting an elevated risk, while those under 1 year showed a substantially reduced risk. The breed analysis indicated that crossbred animals were significantly more susceptible to LSD and exotic breeds displayed a slightly increased risk compared to indigenous cattle. Gender differences were also apparent, as females demonstrated a higher risk compared to males, suggesting a mild predisposition. Cattle in the unorganised farm showed an elevated risk of contracting LSD than the organised farms.

### 3.4. LSD Positivity and Its Association with Other Disease Conditions

Among the LSD-positive animals, 12.6% were also positive for babesiosis, highlighting a moderate prevalence of this haemoprotozoan infection. Anaplasmosis was detected in 24.7% of the LSD-positive cases, suggesting a stronger association and potential for concurrent infections. *Theileria orientalis* was identified in 50.9% of the LSD-positive animals, representing the highest comorbidity rate among the pathogens studied. *T. annulata* was found in 15.4% of the LSD-positive animals, further contributing to the complexity of comorbid conditions. Additionally, 34.1% of the LSD-positive cattle were also positive for infectious bovine rhinotracheitis (IBR), while 12.1% were affected by malignant catarrhal fever (MCF).

**Figure 5:**
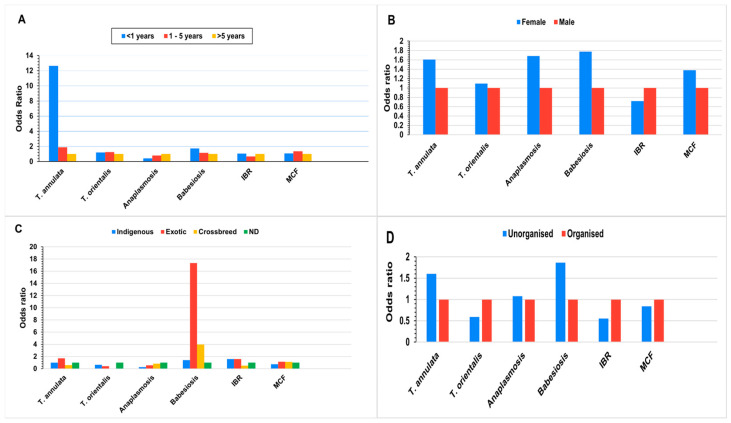
Risk of comorbidity in LSD. The univariate regression analysis showing different diseases and their associated risk factors (age: (**A**); gender: (**B**); breed: (**C**); and farm type: (**D**) in LSD-positive cases.

Among all the 214 LSD-positive animals, the association of risk factors with each disease condition were analysed by regression analysis. In anaplasmosis, statistical significance was observed for breed and age as risk factors in the univariate analysis (Figure 5), but age became non-significant in the subsequent multivariate analysis (Table 3). However, ND females in unorganised farms of over 5 years of age exhibited a higher risk for anaplasmosis compared to other parameters within the same risk group.

The incidence of tropical theileriosis caused by *T. annulata* and *T. orientalis* showed non-significant dependence on the risk factors in both regression analyses. However, *T. annulata* was found more in exotic female cattle less than 1 years of age in unorganised farms, while non-descript females age group between 1 to 5 years in organised farms revealed greater susceptibility to *T. orientalis*.

Babesiosis exhibited a significant association in both the univariate and multivariate analyses. Among the different breeds, exotic cattle demonstrated a 17- to 22-fold increased risk for babesiosis compared to other breeds within the group. Additionally, in both analyses, female calves under one year of age from unorganised farms showed a higher susceptibility to the disease compared to their counterparts with the same parameters.

With regard to IBR, both univariate and multivariate analyses indicated no significant association with the evaluated risk factors. However, in both analyses, exotic male calves under one year of age from organised farms exhibited a higher susceptibility to IBR compared to other groups.

For MCF, none of the assessed risk factors demonstrated statistical significance (Table 3 and Supplementary Table S1). However, cattle aged 1 to 5 years exhibited an increased risk of LSD. Regarding breed susceptibility, exotic cattle (OR: 1.154; CI: 0.329–4.053) and crossbreeds (OR: 1.11; CI: 0.244–5.055) had a higher likelihood of infection. Additionally, females showed 37% to 39% susceptibility to MCF compared to males, as illustrated in Figure 5B. Cattle from organised farms exhibited greater risk to MCF compared to those from unorganised farms.

### 3.5. LSD Negativity and Its Association with Other Disease Conditions

Out of 200 animals that tested negative for LSD, 25.8% were positive for anaplasmosis, while 9.3% were positive for babesiosis. Regarding theileriosis, *Theileria annulata* was detected in 17.4% of cases, whereas *Theileria orientalis* was identified in 40.6% of samples. Infectious bovine rhinotracheitis (IBR) was detected in 35.2% of animals, and malignant catarrhal fever (MCF) was observed in 20% of cases. A regression analysis was performed to assess the association of risk factors with each disease among all 200 LSD-negative samples.

In the case of anaplasmosis, all the evaluated risk factors demonstrated statistical significance in the univariate analysis (Supplementary Table S1). However, in the multivariate analysis, breed and farm type did not retain statistical significance (Supplementary Table S2). The indigenous breeds exhibited a 3.2- to 6-fold higher susceptibility to the disease, followed by exotic and crossbreeds, when compared to non-descript cattle. Additionally, male cattle older than five years showed a higher likelihood of anaplasmosis, particularly in organised farming systems.

Theileriosis caused by *Theileria annulata* and *Theileria orientalis* demonstrated a significant association with all the evaluated risk factors in the univariate analysis. However, in the multivariate analysis, only age lost statistical significance. For *T. annulata*, females exhibited a lower risk than males, whereas cattle younger than one year showed 2.7 to 5 times higher susceptibility to the disease. Additionally, exotic cattle had a 5- to 8-fold increased risk of infection compared to other breeds within the same group. Regarding *T. orientalis*, non-descript cattle under one year of age displayed lower susceptibility compared to other counterparts, while females had reduced susceptibility to infection. In both cases, cattle raised on organised farms exhibited a higher risk of infection compared to those on unorganised farms.

For babesiosis, only farm type revealed significant dependency in the univariate analysis, which became non-significant in the multivariate analysis along with other risk factors. Exotic males aged less than one year in unorganised farms exhibited more susceptibility to the disease.

For infectious bovine rhinotracheitis (IBR), age (*p* = 0.033) was identified as a potential risk factor in the univariate analysis (Supplementary Table S1), with exotic males aged 1 to 5 years in organised farms exhibiting a higher likelihood of infection.

In the case of malignant catarrhal fever (MCF), age did not show statistical significance in the univariate analysis, and in the multivariate analysis, none of the evaluated risk factors remained significant. However, crossbred male cattle under one year of age in unorganised farms demonstrated a higher risk of MCF compared to other parameters within the same group.

### 3.6. LSD and Comorbidity

The chi-square analysis (Table 4) demonstrated a significant association between lumpy skin disease (LSD) and coinfection with other diseases (χ^2^ = 133.1; *p* < 0.0001), with *Theileria orientalis* exhibiting the strongest correlation. Notably, 51% of the LSD-positive animals were concurrently infected with *T. orientalis*, while 34% harboured infectious bovine rhinotracheitis (IBR).

Additionally, the co-occurrence of LSD with two infectious agents (*T. orientalis* and IBR) was statistically significant (χ^2^ = 151; *p* < 0.0001), with 20% of LSD-infected cattle displaying this comorbid condition. The association remained significant when LSD was present alongside three infectious agents (*T. orientalis*, anaplasmosis, and IBR) (χ^2^ = 32.35; *p* = 0.004). However, the presence of LSD in conjunction with four or five infectious diseases did not show statistical significance.

### 3.7. Vector Transmission and Comorbidity

**Figure 6:**
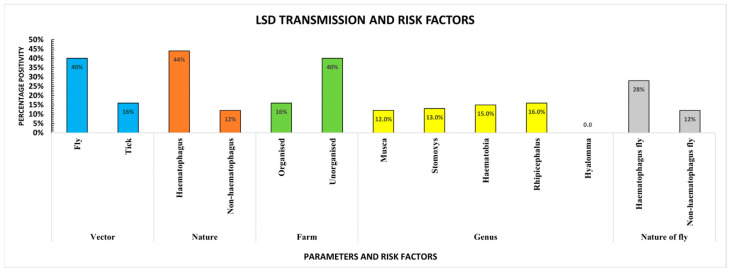
Transmission of LSD and risk factors. The chi-square analysis showing transmission of LSD and its associated risk factors.

The chi-square analysis indicated a significant association between vectors, particularly flies (χ^2^ = 143.3; *p* < 0.0001), and the transmission of an infectious pathogen alongside lumpy skin disease (LSD). Among these, *Theileria orientalis* exhibited the strongest correlation. In contrast, ticks (χ^2^ = 17.33; *p* = 0.0081) were significantly associated with the transmission of one, two, and three diseases. Anaplasmosis showed the highest prevalence in single-disease transmission, whereas anaplasmosis and babesiosis were the most commonly co-transmitted diseases. The simultaneous transmission of *T. orientalis*, anaplasmosis, and babesiosis was observed in cases involving three diseases. In the case of vectors, hematophagous flies in unorganised farms, where *Haematobia* was present, showed higher LSD transmission (Figure 6).

Regarding vector type, hematophagous flies demonstrated a significant association (χ^2^ = 99.47; *p* < 0.0001), with *T. orientalis* being the most frequently transmitted pathogen in single-disease cases. A similar trend was observed for the transmission of one, two, and three diseases alongside LSD, mirroring the pattern seen in ticks (Supplementary Table S3).

Farm management practices also played a significant role. Unorganised farms exhibited a strong association (χ^2^ = 57.91; *p* < 0.0001) with the transmission of *T. orientalis* alongside LSD, following a trend similar to tick-borne transmission patterns for one, two, and three diseases. Organised farms also demonstrated statistical significance (χ^2^ = 39.32; *p* < 0.0001) in the transmission of *T. orientalis* alongside LSD.

At the genus level, *Haematobia* flies showed the highest transmission of *T. orientalis* (χ^2^ = 105.0; *p* < 0.0001). Among ticks, *Rhipicephalus* exhibited a significant association (χ^2^ = 17.33; *p* = 0.0081), following a transmission pattern similar to that of ticks overall.

Finally, the nature of flies was a key factor, with hematophagous flies displaying a statistically significant association (χ^2^ = 143.3; *p* < 0.0001) that mirrored the general transmission pattern observed in flies. However, the vector did not show any transmission of the multiple pathogens in the absence of the LSD.

The LSDV-positive vector samples exhibited geographical overlap with the percentage positivity of LSDV detected in the blood samples collected from outbreak regions. The highest positivity was observed in Bangalore Rural, where case fatality reached 31.26% during the 2022–2023 period. The percentage positivity in the blood samples and vector samples was recorded at 21% and 12%, respectively. A similar trend was observed across 10 other districts in Karnataka with overall positivity in the blood sample and vector sample.

## 4. Discussion

Even though reports regarding the prevalence of LSD, MCF, IBR, and haemoprotozoan infections in cattle in India are available, to the best of the authors’ knowledge, no study has yet investigated the coexistence of these infections in susceptible animals. This gap in the research leaves a critical understanding of the potential interactions between these pathogens and their collective impact on cattle health unexplored. Hence, this study was undertaken to investigate the coexistence of these infections in susceptible cattle in order to identify the risk factors and transmission route contributing to the co-occurrence of these diseases.

Lumpy skin disease (LSD) is a notifiable viral transboundary disease affecting cattle, recognised by the OIE, and has become a global concern due to its rapid spread and substantial socio-economic impact [2]. The risk factors for LSD are diverse, encompassing environmental conditions, management practices, and livestock-specific traits, such as age, sex, breed, and vector prevalence. LSDV transmission is primarily mediated by blood-feeding vectors, including ticks and biting flies, which not only spread LSD but also act as vectors for haemoprotozoan diseases such as theileriosis (*T. annulata* and *T. orientalis*), babesiosis, and anaplasmosis, resulting in frequent coinfections [7]. Biting flies like *Stomoxys* and *Haematobia* have demonstrated high efficiency in transmitting *T. orientalis* and LSDV. Incorporating these factors into risk analysis is crucial for understanding disease dynamics and implementing targeted prevention and control measures.

Out of the 414 samples screened for multiple infectious diseases, 214 (51.6%) tested positive for LSD, reaffirming its significant prevalence among the affected cattle. Among the haemoprotozoan infections, babesiosis was detected in 48 samples (10.95%), while anaplasmosis was observed in 111 samples (25.3%). Theileriosis caused by *T. orientalis* was the most prevalent haemoprotozoan infection, with 200 samples (45.6%) testing positive, whereas infection with *T. annulata* was detected in 72 samples (16.43%). Additionally, viral infections were also widespread, with 152 (34.7%) samples positive for IBR and 71 (16.2%) for MCF. These findings suggest a substantial burden of coinfections in LSD-affected animals, particularly with *T. orientalis*, which exhibited a high degree of co-occurrence with LSD. Jameel (2016) [26] reported coinfections among 50 LSD-infected cattle, with 3.5% having *Babesia*, 4.5% having *Theileria*, and 2% having *Anaplasma*, highlighting the potential for multiple infections in affected animals. In our study, among 176 LSD-positive cattle, coinfections were observed, with 56.3% positive for *Theileria orientalis*, 30.1% for IBR, 28.4% for anaplasmosis, 18.8% for *Theileria annulata*, 14.2% for babesiosis, and 13.6% for MCF. The higher rate of concurrent infections in the present study may be attributed to the high prevalence of ticks and biting flies in the study area as well as the non-availability of any national-level control programs targeting the studied diseases in India. However, this finding should be interpreted cautiously, as the simultaneous injection of multiple pathogens by a tick into a single animal can result in severe clinical outcomes. The presence of multiple infectious agents could exacerbate disease severity and complicate clinical management, making effective treatment more challenging.

In our study, we focused on assessing the effect of risk factors such as age, breed, and sex on the disease incidence among the tested animals. A univariate analysis revealed that females more than 5 years are more susceptible to LSD, likely due to the chance of increased exposure to vectors that transmit the disease. In female animals, physiological stress due to repeated pregnancies, lactation, and hormonal fluctuations may also compromise immune function, increasing their vulnerability to LSD. Moreover, older lactating females often receive more frequent handling and movement, which could inadvertently expose them to higher vector densities in farm environments. This finding aligns with the observations of Odonchimeg et al. (2022) [27], with a slight deviation in the multivariate analysis where lower risk was observed among adults aged 2.5 to 4 years, whereas our study found a consistent risk group among female cattle of more than 5 years of age. Reddy et al. (2024) [28] previously reported the highest incidence of LSD in female cattle (70.53%) and those older than five years. The breed-wise analysis indicated that crossbreed cattle have lower resistance to LSD, consistent with the findings of Hasib et al. (2021). This may be due to the fact that crossbred cattle are frequently raised in intensive or commercial systems where management practices might not be as rigorous, potentially leading to higher stress levels and increased susceptibility to diseases, including LSD.

LSDV’s immune-suppressive effects contribute to the increased susceptibility of cattle to concurrent infections. The virus induces leukopenia and significant immunosuppression, particularly in the later stages of the disease [29,30]. This immune suppression reduces macrophage activity, crucial for controlling intra-erythrocytic haemoprotozoan infections, and diminishes Th1 cytokine production, including gamma interferon (IFN-α) and tumor necrosis factor-alpha (TNF-α), exacerbating haemoprotozoal infections [31]. Moreover, the animals, once infected and recovered from haemoprotozoan parasitic infections, can still harbour low levels of persistent infection in their body and become clinically ill when their immunity wanes due to stress or coinfection with other pathogens [11,32,33]. Thus, during viral infections like LSD, pre-existing haemoprotozoan infections can flare up, a situation aggravated by the presence of tick vectors. The coinfections with haemoprotozoan parasites can exacerbate LSD outcomes by intensifying immunosuppression, prolonging disease duration, increasing susceptibility to secondary bacterial infections, and contributing to greater economic losses in affected cattle [30].

Flies, particularly *Stomoxys*, *Haematobia*, and *Musca*, reproduce in moist, decomposing organic matter, including rotting hay and manure. Poor hygiene conditions in unorganised farms, such as accumulated manure and prolonged storage of feed, contribute to increased disease transmission, as observed in our findings. Our study identified a strong correlation between LSD and coexisting haemoprotozoan diseases and viral infections, supported by a chi-square value of 133.1 (*p* < 0.0001). Transmission studies also highlighted the role of vectors, particularly biting flies like *Stomoxys* and *Haematobia*, as significant mechanical vectors for *Theileria orientalis* (theileriosis) and LSDV [14,34]. *Musca domestica* was also implicated in LSDV transmission, likely due to its interaction with open wounds or scabs created during treatment, which provide a source for blood meals [35]. Hard ticks, such as *Rhipicephalus* spp., were found to transmit LSDV and different haemoprotozoan diseases. However, biting flies demonstrated a greater capacity for disease transmission compared to ticks. This association underscores the potential role of vector-borne haemoprotozoan diseases as significant risk factors for LSD. A previous report by Chihota et al. (2001) [8] underscores the role of ticks in spreading diseases like theileriosis and anaplasmosis, which are prevalent in LSD outbreak regions. The presence of multiple infections in LSD-positive animals highlights the need for integrated disease management strategies, including comprehensive vector control measures. While the detection of multiple pathogens in vectors and cattle does not confirm simultaneous transmission of pathogens by the same vector, the possibility of co-transmission remains, highlighting the complex role of vectors in disease dynamics.

In our study, we observed that among cattle infected with LSD, females were generally at a higher risk of concurrent infections compared to males, with the exception of IBR.

The findings are in concordance with the results of Kumar et al., 2023 [36], who also reported a higher incidence of babesiosis in female cattle. Indigenous breeds exhibited greater resistance to most of the diseases, whereas crossbred cattle showed increased susceptibility. This aligns with previous reports suggesting that crossbred animals, due to their higher production potential, are more vulnerable to diseases, likely due to the stress associated with their higher metabolic demands [17,37]. In the case of anaplasmosis and oriental theileriosis, non-descript breeds showed increased susceptibility to infection. This may be due to the low immunity level in non-descript cattle and the practice of rearing non-descript cattle in a free-grazing system, leading to higher exposure to ticks and biting flies. Further, in the present study, LSD cases were more common in cattle reared in unorganised farms. The higher prevalence of LSD in cattle reared on unorganised farms may be due to poor biosecurity measures, inadequate vector control, and higher animal movement. In LSD-positive animals, oriental theileriosis, IBR, and MCF were observed more in cattle reared in organised farms, whereas anaplasmosis, babesiosis, and bovine tropical theileriosis were more common in those from unorganised farms.

Interestingly, in cattle not infected with LSD, females exhibited greater resistance to haemoprotozoan parasites (*T. orientalis*, *T. annulata*, anaplasmosis, and babesiosis), while males showed higher resistance to MCF. This pattern indicates that the immunosuppression induced by the LSD virus can increase the susceptibility of animals to haemoprotozoan parasites. Previous reports [28] also suggest that a higher prevalence of tick-borne haemoparasites was detected in LSD-positive animals. Indigenous breeds showed lower infection rates for all diseases in LSD-negative animals, except for anaplasmosis and theileriosis.

Our findings revealed significantly higher incidence rates of haemoprotozoan parasites, as well as IBR and MCF, in LSD-positive cattle compared to those that were LSD-negative. This comprehensive risk factor analysis offers valuable insights for veterinarians, aiding in the understanding of disease dynamics and facilitating the development of more effective treatment strategies. It highlights the importance of considering the broader ecological and epidemiological context, particularly in regions where vectors such as flies and ticks play a significant role in the transmission of multiple diseases. The use of attenuated vaccines presents certain limitations and risks. Therefore, a comprehensive disease control strategy that integrates vector management is recommended. Furthermore, while we established the presence of different pathogens in vectors and cattle, a geographic analysis of their distribution could provide valuable insights into regional hotspots of vector-borne coinfections. Future studies should focus on evaluating the clinical implications of these coinfections, assessing their impact on LSD progression, and developing integrated disease management strategies that target both LSD and associated vector-borne pathogens. Our study lays a strong foundation for future research into comorbidities associated with bovine diseases and their transmission. However, further investigation is necessary to fully understand the intricate interactions between these diseases and their vectors.

## Figures and Tables

**Table 1:** Details of primers used. The table showing the details of the primers (nucleotide sequence, annealing temperature, and amplicon size) used for confirmation of LSD, IBR, MCF, babesiosis, anaplasmosis, and theileriosis (*Thileria orientalis* and *Thileria annulata)* by PCR.

S.NO	Disease	Primer	Sequence (5′-3′)	Base Pair	Annealing Temperature	Reference
1	LSD	SGPP32FP	ACACAGGGGGAT ATGATTTTACC	237 bp	52 °C	[18]
		SGPP32RP	ATACCGTTTTTC ATTTCGTTAGC			
2	Theileriosis (*Thileria orientalis*)	ToMPSP-F	CTTTGCCTAGGATACTTCCT	776 bp	60 °C	[19]
		ToMPSP-R	ACGGCAAGTGGTGAGAACT			
3	Theileriosis (*Thileria annulata*)	TaCytb-F	ACTTTGGCCGTAATGTTAAAC	312 bp	64 °C	[20]
		TaCytb-R	CTC TGGACCAACTGTTTGG			
4	Anaplasmosis	Ana Common-F	GCTGTTCCTAGGCTYTCTTACGCGA	525 bp	55 °C	[21]
		Ana Common-R	AATCRAGCCAVGAG CCCCTRTAWGG			
5	Babesiosis	B Common-F	GCATTTGCGATGGACCATTCAAG	200 bp	57 °C	[22]
		B Common-R	CCTGTATTGTTATTTCTTGTCACTACCTC			
6	IBR	gBF	TGGTGGCCTTYGACCGCGAC	293 bp	58 °C	[23]
		gBR	GCTCCGGCGAGTAGCTGGTGTG			
7	MCF	775	AAGATAAGCACCAGTTATGCATCTGATAAA	422 bp	60 °C	[24]
		556	AGTCTGGGGTATATGAATCCAGATGGCTCTC			

**Table 2:** Multivariate analysis of LSD risk factors. Multivariate logistic regression analysis highlighting the association between risk factors (breed, sex, age, and type of farm) and lumpy skin disease (LSD).

Risk Factors	Parameters	Sig.	OR	95% CI	
				Lower	Upper
Breed	Indigenous	0 *	0.452	0.244	0.836
	Exotic		0.920	0.507	1.671
	Crossbreeds		3.366	1.703	6.656
	ND		1	-	-
Sex	Female	0.172	1.455	0.85	2.490
	Male	-	1	-	-
Age	<1 years	0 *	0.172	0.093	0.32
	1–5 years		0.552	0.329	0.925
	>5 years		1	-	-
Farm	Unorganised farm	0 *	1.708	1.170	2.494
	Organised farm	-	1	-	-

Abbreviations: Sig. = significant (* *p* < 0.05 = significant), OR = odds ratio, and CI = confidence interval.

**Table 3:** LSD risk factors and disease association. Comprehensive multivariate logistic regression analysis of risk factors (breed, sex, age, and farm type) for lumpy skin disease (LSD) positivity and its association with different cattle’s disease.

Risk Factors		Breed				Sex		Age			Farm	
Comorbidities		Indigenous	Exotic	Crossbreeds	ND	Female	Male	<1 Year	1–5 Years	>5 Years	Unorganised	Organised
**Oriental theileriosis**	**Sig.**	0.427				0.813	1	0.538			0.073	1
	**OR**, **95% CI**	0.724 (0.334–1.57)	0.472 (0.194–1.143)	0.643 (0.226–1.831)	1	1.095 (0.515–2.332)		0.582 (0.244–1.511)	1.068 (0.514–2.221)	1	0.458 (0.1951.075)	
**Tropical theileriosis**	**Sig.**	0.079				0.644	1	0.706			0.435	1
	**OR**, **95% CI**	0.712 (0.231–2.195)	3.573 (0.918–13.899)	1.051 (0.272–4.057)	1	1.055 (0.3–2.105)		1.746 (0.41–7.433)	1.571 (0.465–5.314)	1	1.605 (0.489–5.264)	
**Anaplasmosis**	**Sig.**	0.005 *				0.397	1	0.047 *			0.069	1
	**OR,** **95% CI**	0.255 (0.096–0.672)	0.632 (0.217–1.838)	0.987 (0.253–3.853)	1	1.361 (0.667–2.77)		0.421 (0.18–0.983)	0.886 (0.355–2.21)	1	1.768 (0.956–3.344)	
**Babesiosis**	**Sig.**	0.029 *				0.469	1	0.706			0.622	1
	**OR**, **95% CI**	1.316 (0.511–3.390)	21.885 (2.456–194.978)	4.45 (0.791–25.02)	1	1.778 (0.76–4.16)		1.746 (0.41–7.453)	1.571 (0.465–5.314)	1	1.368 (0.395–4.739)	
**IBR**	**Sig.**	0.5				0.819	1	0.538			0.125	1
	**OR**, **95% CI**	1.613 (0.718–3.623)	2.015 (0.789–5.142)	0.737 (0.255–2.127)	1	0.912 (0.413–2.012)		1.055 (0.499–2.232)	0.69 (0.318–1.497)	1	0.492 (0.199–1.218)	
**MCF**	**Sig.**	0.824				0.564	1	0.834			0.313	1
	**OR**, **95% CI**	0.737 (0.242–2.247)	1.154 (0.329–4.053)	1.111 (0.244–5.055)	1	1.395 (0.45–4.332)		1.085 (0.392–3.002)	1.372 (0.465–4.051)	1	0.519 (0.145–1.856)	

Abbreviations: Sig. = significant (* *p* < 0.05 = significant), OR = Odds ratio and CI = confidence interval.

**Table 4:** Disease incidence in cattle. Incidence of haemoprotozoan parasites, IBR, and MCF in LSD-positive and LSD-negative cattle with statistical comparison.

Parameters	Comorbidities	LSD Positive			LSD Negative		
		Chi-Square, df	Significance	Percentage	Chi-Square, df	Significance	Percentage
**Comorbidity with one disease**	***T. orientalis***	133.1, 5	<0.0001 *	51%	86.45, 5	<0.0001 *	41%
	***T. annulata***			15%			17%
	**Anaplasmosis**			25%			26%
	**Babesiosis**			13%			12%
	**IBR**			34%			33%
	**MCF**			12%			12%
**Comorbidity with two diseases**	***T. orientalis+* Anaplasmosis**	151.0, 14	<0.0001 *	19%	151.6, 14	<0.0001 *	18%
	***T. orientalis+* Babesiosis**			7%			5%
	***T. orientalis*+ IBR**			20%			18%
	***T. orientalis*+ MCF**			2%			7%
	***T. orientalis+ T. annulata***			6%			9%
	***T. annulata+* Anaplasmosis**			2%			6%
	***T. annulata+* Babesiosis**			2%			2%
	***T. annulata+* IBR**			7%			7%
	***T. annulata+* MCF**			3%			2%
	**Anaplasmosis+ IBR**			10%			8%
	**Anaplasmosis+ MCF**			3%			4%
	**Anaplasmosis+ Babesiosis**			10%			1%
	**IBR+ MCF**			5%			11%
	***IBR+* Babesiosis**			3%			1%
	***MCF+* Babesiosis**			3%			1%
**Comorbidity with three diseases**	***T. orientalis+* Anaplasmosis+ Babesiosis**	32.35, 10	0.0004 *	4%	29.11, 10	0.0012 *	1%
	***T. orientalis+* Anaplasmosis*+ T. annulata***			2%			4%
	***T. orientalis*+ Anaplasmosis+ IBR**			7%			6%
	***T. orientalis*+ Anaplasmosis+ MCF**			2%			4%
	***T. orientalis+* Babesiosis*+ T. annulata***			1%			1%
	***T. orientalis*+ Babesiosis+ IBR**			1%			1%
	***T. orientalis*+ Babesiosis+ MCF**			1%			1%
	***T. orientalis+* IBR*+ T. annulata***			5%			6%
	***T. orientalis*+ IBR+ MCF**			5%			6%
	***T. orientalis*+ *T. annulata*+ MCF**			3%			2%
**Comorbidity with four diseases**	**Babesiosis+ Anaplasmosis+ *T. orientalis+ T. annulata***	0.000, 2	>0.9999	0.5%	2.009, 2	0.3662	0.9%
	**Babesiosis+ Anaplasmosis+ *T. orientalis*+ IBR**			0.5%			0.4%
	**Babesiosis+ Anaplasmosis+ *T. orientalis*+ MCF**			0.5%			0.0%
**Comorbidity with five diseases**	**Babesiosis+ Anaplasmosis+ *T. orientalis*+ MCF+ *T. annulata*+ IBR**	-----		0.5%	-----		0.0%

* Significance = *p* < 0.05, significant.

## Data Availability

The data that support the findings of this study are available from the corresponding author upon reasonable request.

## Supplementary Materials

The following supporting information can be downloaded at: https://www.mdpi.com/article/10.3390/microorganisms13030472/s1, Table S1: LSD Risk Factors with Disease and Demographics; Table S2: Risk Factors and Disease Analysis in Cattle; Table S3: Transmission of LSD and Disease Association.
