# Supplementary material for: Investigation of Comorbidity and Risk Factors Analysis During Lumpy Skin Disease Outbreaks in India
Source: Microorganisms. 2025 Feb 20;13(3):472. doi: 10.3390/microorganisms13030472 (PMC11946313; doi:10.3390/microorganisms13030472)
Supplement: Supplementary file 1 [file microorganisms-13-00472-s001.zip › microorganisms-3445925-supplementary.pdf]

Supplementary Table S1 LSD Risk Factors with Disease and Demographics. Univariate Regression Analysis of Risk Factors Associated with LSD-Negative Cattle, Including Theileriosis (*T. annulata and T. orientalis*), Anaplasmosis, Babesiosis, IBR, and MCF, Stratified by Age, Breed, Sex and farm type.

RISK FACTORS		Breed				Sex		Age			Farm	
**COMORBITIES**		**Indigenous**	**Exotic**	**Crossbreeds**	**ND**	**Female**	**Male**	**<1 year**	**1-5 years**	**>5 years**	**Unorganised**	**Organised**
**Oriental theileriosis**	**Sig.**	**0.001***				**0.021***	1	**0.028***			**0.011***	1
	**OR, 95% CI**	4.891 (2.011-11.898)	1.656 (0.752-3.648)	1.025 (0.489-2.147)	1	0.52 (0.298-0.907)		0.371 (0.178-0.776)	0.592 (0.318-1.104)	1	0.494 (0.287-0.849)	
**Tropical theileriosis**	**Sig.**	**0.003***				**0.0***	1	**0.045***			**0.0***	1
	**OR, 95% CI**	4.083 (1.445-11.537)	8.33 (2.247-30.901)	1.929 (0.833-4.467)	1	0.166 (0.62-0.442)		4.839 (1.355-17.277)	1.573 (0.752-3.387)	1	0.154 (0.067-0.353)	
**Anaplasmosis**	**Sig.**	**0.012***				**0.003***	1	**0.005***			0.062	1
	**OR, 95% CI**	5.795 (1.769-18.987)	0.884 (0.384-2.035)	1.017 (0.458-2.26)	1	0.359 (0.183-0.705)		0.258 (0.109-0.612)	0.347 (0.161-0.751)	1	0.564 (0.309-1.03)	
**Babesiosis**	**Sig.**	0.235				0.4	1	0.37			0.019*	1
	**OR, 95% CI**	1.707 (0.504-5.781)	8.878 (1.05-75.075)	1.28 (0.431-3.806)	1	0.307 (0.1-0.946)		2.486 (0.506-12.225)	0.816 (0.311-2.317)	1	2.328 (1.147-4.726)	
**IBR**	**Sig.**	0.308				0.442	1	**0.033***			0.442	1
	**OR, 95% CI**	0.646 (0.289-1.441)	1.393 (0.592-3.279)	0.86 90.396-1.87)	1	1.305 (0.465-1.398)		2.097 (0.982-4.479)	2.143 (1.15-3.995)	1	0.806 (0.465-1.398)	
**MCF**	**Sig.**	**0.0***				**0.002***	1	0.085			**0.019***	1
	**OR, 95% CI**	0.238 (0.091-0.623)	0.960 (0.320-2.883)	1.488 (0.485-4.56)	1	2.96 (1.506-5.817)		3.153 (1.107-8.984)	1.548 (0.762-3.146)	1	2.328 (1.147-4.726)	

Abbreviations: Sig. = Significant(*p<0.05=significant), OR = Odds Ratio, CI = Confidence Interval.

Supplementary Table S2 Risk Factors and Disease Analysis in Cattle. Multivariate Regression Analysis of Diseases and Risk Factors, Including Theileriosis (*T. annulata and T. orientalis*), Anaplasmosis, Babesiosis, IBR, and MCF, in LSD-Negative Cattle Stratified by Age, Breed, Sex and farm type.

**RISK FACTORS**		**Breed**				**Sex**		**Age**			**Farm**	
**COMORBITIES**		**Indigenous**	**Exotic**	**Crossbreeds**	**ND**	**Female**	**Male**	**<1 year**	**1-5 years**	**>5 years**	**Unorganised**	**Organised**
**Oriental theileriosis**	**Sig.**	0.064				0.293		**0.021***			0.254	
	**OR, 95% CI**	3.484 (1.324-9.17)	2.351 (0.946-5.842)	1.387 (0.618-3.113)	1	0.677 (0.327-1.401)	1	0.265 (0.104-0.68)	0.669 (0.338-1.326)	1	0.617 (0.346-1.323)	1
**Tropical theileriosis**	**Sig.**	**0.027***				0.677		0.352			**0.002***	
	**OR, 95% CI**	1.275 (0.356-4.562)	5.298 (1.25-22.456)	3.664 (1.397-9.61)	1	0.184 (0.059-0.571)	1	2.682 (0.65-11.067)	1.441 (0.624-3.326)	1	0.203 (0.073-0.564)	1
**Anaplasmosis**	**Sig.**	0.179				**0.01***		**0.01***			0.279	
	**OR, 95% CI**	3.235 (0.901-11.617)	1.041 (0.399-2.718)	1.730 (0.719-4.163)	1	0.317 (0.132-0.76)	1	0.198 (0.069-0.569)	0.414 (0.18-0.952)	1	0.663 (0.315-1.394)	1
**Babesiosis**	**Sig.**	0.183				**0.029***		0.642			**0.005***	
	**OR, 95% CI**	2.39 (0.581-9.834)	10.724 (1.137-92.831)	1.418 (0.427-4.716)	1	0.225 (0.059-0.861)	1	2.376 (0.392-14.397)	1.298 (0.405-4.155)	1	5.721 (1.7-19.252)	1
**IBR**	**Sig.**	0.67				0.397		0.136			0.358	
	**OR, 95% CI**	0.678 (0.271-1.694)	1.053 (0.401-2.765)	0.729 (0.315-1.448)	1	1.361 (0.667-2.777)	1	1.773 (0.718-4.38)	1.932 (0.997-3.742)	1	0.721 (0.359-1.448)	1
**MCF**	**Sig.**	0.17				0.062		0.19			0.656	
	**OR, 95% CI**	0.328 (0.112-0.959)	0.681 (0.192-2.413)	1.248 (0.295-3.346)	1	2.276 (0.959-5.401)	1	3.093 (0.816-11.731)	1.353 (0.434-2.2)	1	1.222 (0.505-2.958)	1

Abbreviations: Sig. = Significant(*p<0.05=significant), OR = Odds Ratio, CI = Confidence Interval.

Supplementary Table S3. Transmission of LSD and Disease Association. Chi square analysis of risk factors for transmission of lumpy skin disease (LSD) positivity and its association with different disease.

**PARAMETERS**	**RISK FACTORS**	**Transmission of one disease**			**Transmission of two diseases**			**Transmission of three diseases**	**Chi-square, df**	**Sig.**
		T. orientalis	**Anaplasmosis**	**Babesiosis**	***Anaplasmosis + Babesiosis***	***T. orientalis + Babesiosis***	***T. orientalis + Anaplasmosis***	***T. orientalis + Anaplasmosis+ Babesiosis***		
**Vector**	Fly	39%	0%	0%	0%	0%	0%	0%	143.3,6	**<0.0001***
	Tick	11%	14%	13%	5%	2%	4%	2%	17.33, 6	**0.0081***
**Nature**	Hematophagous	50%	14%	13%	5%	2%	4%	2%	99.47, 6	**<0.0001***
	Non-hematophagous	0%	0%	0%	0%	0%	0%	0%	-----	
**Farm**	Organised	14%	0%	0%	0%	0%	0%	0%	39.32, 6	**<0.0001***
	Unorganised	36%	14%	7%	5%	2%	4%	2%	57.91, 6	**<0.0001***
**Genus**	*Musca*	0%	0%	0%	0%	0%	0%	0%	-----	
	Stomoxys	13%	0%	0%	0%	0%	0%	0%	49.00, 6	**<0.0001***
	Haematobia	27%	0%	0%	0%	0%	0%	0%	105.0, 6	**<0.0001***
	Rhipicephalus	11%	14%	13%	5%	2%	3%	2%	17.33, 6	**0.0081***
	Hyalomma	0%	0%	0%	0%	0%	0%	0%	-----	
**Nature of fly**	Hematophagous fly	39%	0%	0%	0%	0%	0%	0%	143.3, 6	**<0.0001***
	Non-hematophagous fly	0%	0%	0%	0%	0%	0%	0%	-----	
